# Few-shot recognition of lane-changing intention in connected vehicle environment: A meta-transformer approach

**DOI:** 10.1371/journal.pone.0361594

**Published:** 2026-10-29

**Authors:** Xiaoqian Liu, Wenxin Jiang, Yining Ren

**Affiliations:** 1 School of Business, Xinjiang University, Urumqi, China; 2 School of Transportation, Tongji University, Shanghai, China; 3 The Key Laboratory of Road and Traffic Engineering of the Ministry of Education, Tongji University, Shanghai, China; National University of Singapore, SINGAPORE

## Abstract

This paper proposes a Meta-Transformer model for few-shot accurate lane-changing intention recognition in connected vehicle environment. Given the velocity, acceleration, distance headway, time headway and time to collision of a vehicle at past time steps, a transformer model is developed to recognize the lane changing intention of the vehicle at the current time step. The multi-head attention layer of the Transformer model is capable of effectively extracting features from the multivariate time serie input. This enhances the accuracy of lane-changing intention recognition. The Transformer model is combined with a model -agnostic meta-learning (MAML) algorithm to be able to perform well with small amount of training data. The meta-learning consists of meta-training and meta-testing. During the meta-training, a task is sampled from the distribution over tasks. The tasks include recognition of intention of lane-keeping, lane-changing to left lane, and lane-changing to right lane. The model is trained with few samples and feedback from the loss function defined on the tasks. The loss function is defined as the categorical cross entropy of the errors between the labels and the results generated by the proposed model. During the meta-testing, the model is tested on new data from the distribution of the transition from a feature and the label to another feature. The model is trained by minimizing the test error. Experimental results show that the proposed model can achieve 8.7 percentage points higher accuracy than a transformer model.

## 1 Introduction

Accurately recognizing the lane changing intention of surrounding vehicles is the foundation for the driving safety and efficiency of a vehicle. Lane changing is inevitable and frequent for vehicles. Due to the complication of lane changing, misjudging the lane changing intention of surrounding vehicles may result in additional traffic accidents and/or traffic delay. This is particularly the case for autonomous vehicles [1]. With the aid of vehicle-to-vehicle communication [2], a vehicle can obtain the position and speed of surrounding vehicles in high frequency [3,4]. These high-resolution time series data provide new opportunities for enhancing the accuracy of lane changing intention recognition [5,6]. Recent advances in autonomous driving have increasingly relied on learning-based perception and decision-making. Liang et al. [7] developed a Transformer-transfer learning framework for interaction-aware trajectory prediction in safe motion planning, while integrated deep reinforcement learning has been applied to enhance high-speed cruising performance [8]. These studies highlight the growing demand for accurate and data-efficient intention recognition as a prerequisite for safe autonomous driving.

Previous studies on lane-changing intention recognition can be broadly categorized into hidden-Markov-model (HMM)-based approaches and deep-learning-based approaches. HMM-based methods model the intention as a latent state and infer it from observed motion sequences. Sharma et al. [9] proposed a continuous HMM based on real-world trajectory data, and David et al. [10] developed an improved HMM using simulator data. Liu et al. [5] further introduced a hidden semi-Markov model (HSMM) with a recurrent neural network to capture discrete intention transitions. These methods are interpretable and computationally efficient, but they often rely on hand-crafted state transitions and struggle to capture complex, long-range dependencies in high-resolution trajectory data. Deep-learning-based methods, in contrast, learn representations directly from data. Feng et al. [11,12] used a support vector machine with particle-swarm optimization, Sun et al. [13] proposed a random-forest-based model, Wang et al. [14] combined LSTM with Bayesian inference, and Zhou et al. [15] established a bidirectional LSTM network to extract vehicle motion features. While these methods improve recognition accuracy, they typically require substantial amounts of labeled data to achieve satisfactory performance. As a result, existing approaches still face challenges in simultaneously achieving high accuracy and data efficiency, which motivates the exploration of more expressive and data-efficient sequence models.

Recently, Transformer-based architectures have demonstrated great potential in time-series modeling and have been introduced to lane-changing prediction tasks. Wang et al. [16] incorporated roadside traffic data to predict lane-changing behaviors of automated vehicles within highway weaving segments, leveraging macroscopic traffic state information to enhance microscopic behavior prediction. De Cristofaro et al. [17] systematically compared LSTM, convolutional neural network (CNN), and Transformer networks for human driver lane-change intention prediction using the highD dataset, and found that the Transformer achieved superior performance. These studies confirm the effectiveness of the self-attention mechanism in capturing long-range dependencies and complex interactions among multiple kinematic features. However, Transformer models typically require substantial amounts of training data to achieve satisfactory generalization, which poses a significant challenge for lane-changing intention recognition in practice, where labeled instances of critical lane-change events are rare and costly to annotate.

Meta-learning, also known as few-shot learning, offers a promising solution to the data scarcity problem. First introduced in the 1990s [18], meta-learning aims to learn a generalizable initialization or update rule from a distribution of tasks, enabling rapid adaptation to new tasks with only a few labeled samples. In the transportation domain, meta-learning has recently gained increasing attention. Pan et al. [19] developed a spatio-temporal meta-learning framework for urban traffic prediction. Wei et al. [20] proposed a model-agnostic meta-learning approach for real-time traffic conflict prediction at signalized intersections with limited samples. Kuang et al. [21] applied deep meta-learning to regional parking occupancy prediction by leveraging heterogeneous and real-time information. Beyond transportation, meta-learning has also shown promise in autonomous systems. Sahin and Bakirci [22] developed a few-shot learning framework for vision tasks in autonomous aerial systems under low-light conditions, and evaluated it against established few-shot approaches such as MAML and ProtoNet. In a related but distinct line of work, Bayraktar and Bakirci [23] proposed a decentralized graph-reinforced learning architecture for autonomous airspace coordination under uncertainty, addressing learning-based decision-making under communication delays and sensor uncertainty. These studies demonstrate the value of meta-learning and uncertainty-aware learning in autonomous systems. Despite these successful applications, little attention has been paid to introducing meta-learning into lane-changing intention recognition, particularly in the context of connected vehicle systems where data efficiency is critically important.

To fill this research gap, this study proposes a Meta-Transformer model for few-shot accurate lane-changing intention recognition. The contributions of this work are twofold: (1) A transformer model is developed for lane changing intention recognition;.(2) The transformer model is combined with a Model-Agonestic Meta-learning algorithm to be capable of performing well with small amount of training data.

The rest of this paper is organized as follows. In the following words, Section 2 establishes the transformer model for lane changing intention recognition, and integrates the transformer model with a meta-learning algorithm. The proposed methodology is evaluated in Section 3. Conclusions and further research directions are discussed in Section 4.

## 2 Methodology

This paper assumes that a vehicle maintains a constant lane-changing intention within the recognition horizon. The continuous lane-changing process is represented by a sequence of discrete intention recognition results at successive time steps, rather than by a continuously evolving intention variable. Since motion states such as velocity can reflect the vehicle’s intention, the data reflecting these motion states are used to recognize the lane-changing intention in the following sections.

This study mainly considers three driving maneuvers [24]: Lane-keeping (LK), Lane-changing to left lanes (LCL), Lane-changing to right lanes (LCR). In that way, the recognition of lane-changing intention can be regarded as a multi-classification problem. In order to solve this problem, a Meta-Transformer-based method is developed, which is composed from Transformer and MAML algorithm.

### 2.1 A transformer model for lane-changing intention recognition

The transformer was originally first proposed by Vaswani et al [25]. for natural language processing. It is a model that utilizes attention mechanisms to improve model training speed. In this study, Transformer Network is adopted to recognize the lane-changing intention of vehicles. In order to conduct the experiment of this study conveniently, the intention of LK, LCL and LCR is respectively labelled as 0, 1, 2.

As shown in Fig 1, the Transformer is organized in an encoder-decoder manner, in which identical encoder modules are stacked at the bottom and identical decoder modules are stacked on top. Each encoder module is composed of a multi-head self-attention layer and a position-wise feed forward layer, while each decoder module has one more layer (i.e., encoder-decoder attention layer), which is inserted between the self-attention and feed-forward layers to bridge the encoder and decoder parts. In addition to the two sub-layers in each encoder layer, the decoder inserts a third sub-layer, which performs multi-head attention over the output of the encoder stack.

**Fig 1:**
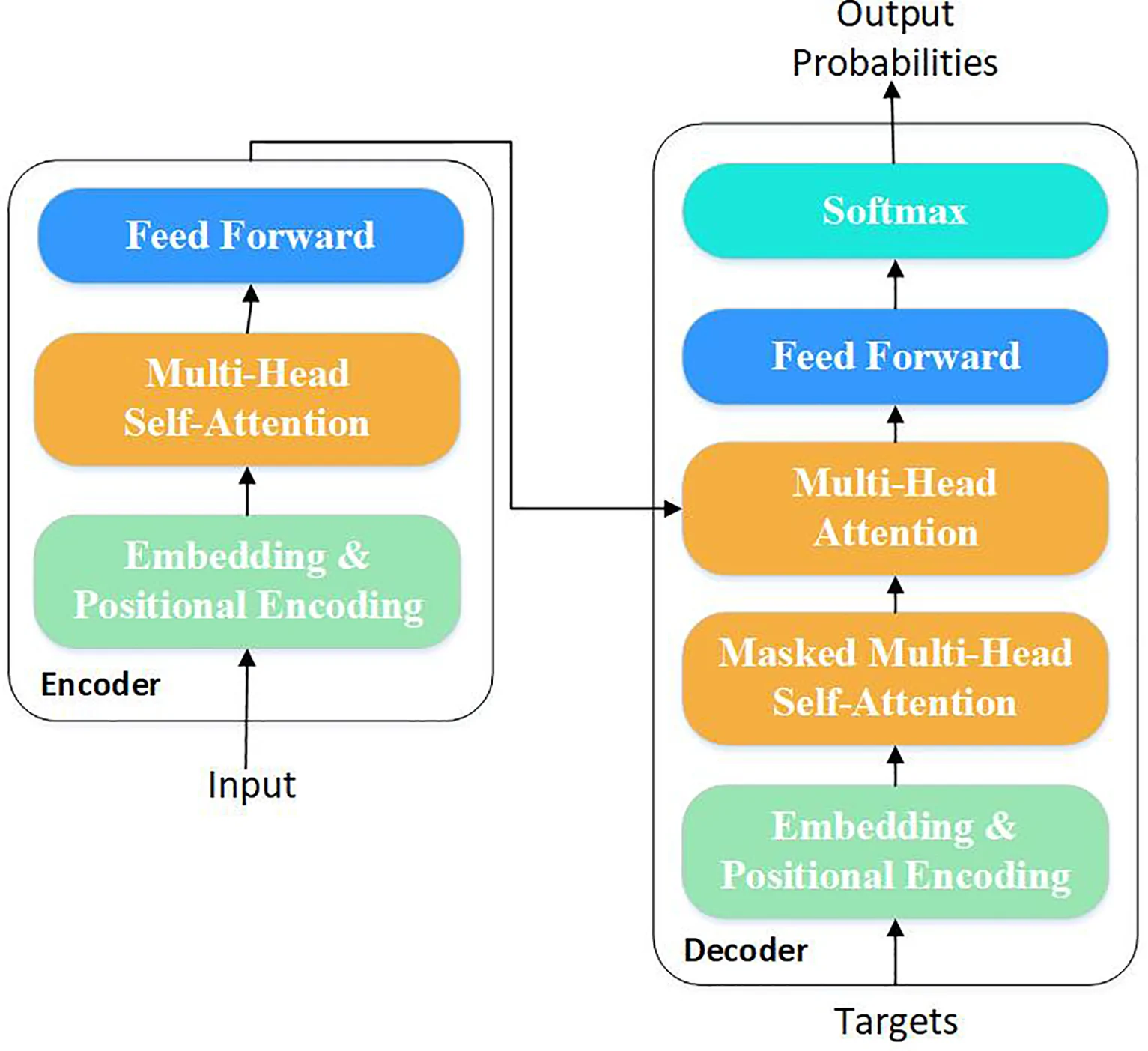
The structure of the transformer model.

The aim of multi-head attention layers is to attach different importance of words/tokens to each other in a sequence from multiple aspects (heads). Then the outputs of those different heads are concatenated and passed through a linear transformation to aggregate all the information. As the attention mechanisms behind the self attention and encoder–decoder attention layers are the same, we take the self-attention layer as an example. It operates on a sequence of tokens $x=\left(x_{\text{1}},x_{\text{2}},\ldots ,x_{n}\right)$, each of which is initialized by a random vector, and is updated by using a weighted sum of any other word after being passed through a linear transformation. The weights, called attention scores, are assigned by their similarities.

Take the update $x_{i}$ as an example:


$$\begin{array}{c} y_{i}=\sum_{j=1}^{n}a_{i,j}\left(x_{j}W_{V}\right) \end{array}$$ (1)

where $y_{i}$ is the update of $x_{i}$,and $a_{i,j}$ is the attention score, measuring the similarity between $x_{i}$ and $x_{j}$, calculated as:


$$\begin{array}{c} a_{i,j}=\frac{exp\left(e_{i,j}\right)}{\sum_{k=1}^{n}exp\left(e_{i,k}\right)} \end{array}$$ (2)

where $e_{i,j}$ measures the compatibility of two linearly transformed $x_{i}$ and $x_{j}$, calculated by using the scaled dot product,


$$\begin{array}{c} e_{i,j}=\frac{\left(x_{i}W_{Q}\right){\left(x_{j}W_{K}\right)}^{T}}{\sqrt{h}} \end{array}$$ (3)

where h is the dimension of the output.Note that $W_{V}$, $W_{Q}$, $W_{K}$are three linear transformation matrices to strengthen the expressiveness of Transformer.

The LC intention is recognized based on the real time information of vehicles, such as speed, acceleration, and other information useful for recognizing the motion of vehicles. The recognition of LC intention can be written as Eq. (4)-(5).


$$\begin{array}{c} \hat{I}=\varphi \left(S\right) \end{array}$$ (4)

$$\begin{array}{c} S\left(t\right)=\left\{x\left(t\right),v\left(t\right),a\left(t\right),\ldots \right\} \end{array}$$ (5)

where $\hat{I}$ represents the intention of lane-changing of vehicles, S(t) represents motion condition series of vehicles. In Eq. (1), $\hat{I}\left(t\right)$ is the function of S(t) which is a sequence of vehicle real-time status, including the position x(t), the velocity v(t), the acceleration a(t) and other status reflecting the movement of vehicles.

During the training process of Transformer, the movement information of vehicles forms a sequence such as S(t) in Eq. (5) which is put into the Transformer Network. The dimension of input is upon the length of sequence put into the Transformer Network. The model will output the recognition results according to the input through the network. The results represent the real-time intention of vehicles. The dimension of output was based on the number of categories.

Considering the recognition of LC intention is regarded as a multi-classification here, we choose categorical crossentropy as the loss function which is shown as Eq. (6)


$$\begin{array}{c} Loss=-\sum_{j=1}^{\text{3}}label_{j}\;log{\hat{p}}_{j} \end{array}$$ (6)

where $label_{j}$ shows the true intention label of vehicles, and ${\hat{p}}_{j}$ represents the estimated probability of each label respectively.

### 2.2 Model-agnostic meta-learning

Model-Agnostic Meta-Learning (MAML) is a meta-learning algorithm proposed in 2017 [26]. It embeds common knowledge from various tasks in the initial parameters of a target model, which can be used to quickly adapt the model to new tasks with few data samples. Benefited from its efficiency of tasks that lack data, MAML is gaining more and more popularity in the regression [27] and classification problem [28]. In this paper, we use MAML to help recognition lane-changing intention, with a particular focus on limited lane-change data for reference.

The process of MAML can be divided into two stages: meta-learning and meta-testing. In the first stage of MAML, meta-learning, several common data samples for various tasks are obtained and embedded as an initial parameters θ in the lane-change intention recognition model. In the second stage of MAML, initial parameters θ are adjusted through a small volume of data samples for target tasks

Fig 2 presents a schematic representation of the MAML algorithm where the initial parameters θ are optimized and fine-tuning for a new target task is conducted in the meta-testing stage. Initial parameters θ are updated twice in the meta-learning stage, first in the process of model training and second in the validation with a few different samples.

**Fig 2:**
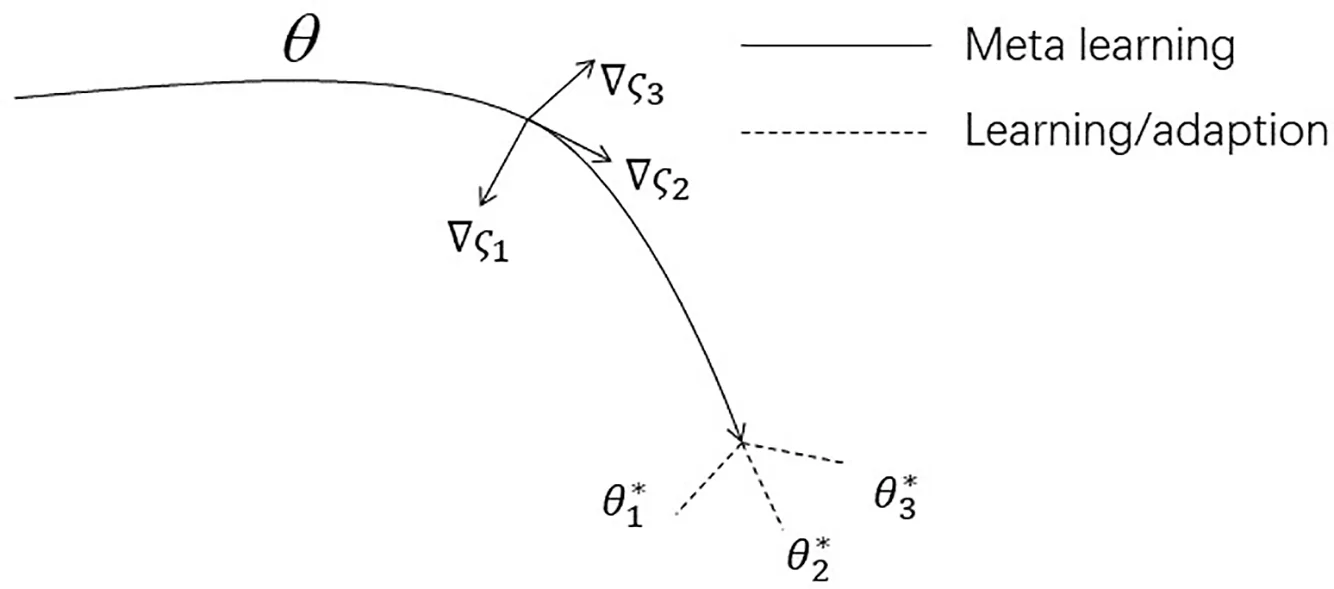
The process of adaption stage in MAML.

The optimization of parameters θ can be regarded as a bi-level optimization associated with inner loop and outer loop of MAML. Let f(θ) be the model to be trained, with parameters θ and the optimization process of parameters θ can be described as follows:

In the meta-training process, θ is evaluated for all meta-tasks and updated in the inner loop of MAML. For each meta-task $\tau_{i}$, MAML computes the adapted parameters $\theta_{i}^{{\;}^{\prime }}$ using the following gradient step:


$$\theta_{i}^{'}=\theta -\alpha \nabla_{\theta }\varsigma_{\tau_{i}}\left(f\left(\theta \right)\right)$$ (7)

where $\varsigma_{\tau_{i}}$is the loss function of task $\tau_{i}$ and α is the learning rate in the inner loop.

Eq. (6) calculates $\theta_{i}^{{\;}^{\prime }}$ which represents the parameters learned for $\tau_{i}$ using the gradient step. Once $\theta_{i}$ has been obtained for each meta-task, θ will be renewed via the formulation described in Eq. (7). In that way, θ is updated and the sum of the loss functions of the meta-tasks is reduced. According to Eq. (8).


$$\theta \leftarrow \theta -\beta \nabla_{\theta }\sum_{i=0}^{M}\varsigma_{\tau_{i}}\left(f\left(\theta_{i}^{'}\right)\right)$$ (8)

where β is the learning rate in the outer loop.

After several epochs of updates, MAML will obtain and output a best parameters θ for the model.

In addition to meta-training and meta-testing, a meta-validation stage is used for model selection and hyperparameter tuning. Tasks in this stage are drawn from a separate validation subset and are not used for updating the meta-parameters.

### 2.3 Task construction and data splitting

The proposed Meta-Transformer framework is trained under the episodic meta-learning paradigm, in which the model learns from a distribution of tasks rather than from a single fixed dataset. In this study, a meta-task is defined as an episodic classification unit containing a support set and a query set, both drawn from the same task distribution. Importantly, the three intention classes (LK, LCL, and LCR) are not treated as separate meta-tasks. Instead, each meta-task contains samples from all three classes, and different meta-tasks differ in the specific vehicle trajectories and driving scenarios from which their support and query samples are drawn. This design reflects the practical requirement that a recognition model must distinguish among all intention classes simultaneously under new driving conditions.

#### 2.3.1 Support and query set construction.

For each meta-task $\tau_{\text{i}}$, a support set $S_{\text{i}}$ and a query set $Q_{\text{i}}$ are sampled from the meta-training pool. The support set is used for inner-loop adaptation, and the query set is used for outer-loop meta-update. To ensure that each episode covers all intention categories, balanced episodic sampling is adopted: the support set contains [N] samples per class, and the query set contains [M] samples per class. In this study, we set [N]=5 and [M]=20 so that each meta-task contains [3N] support samples and [3M] query samples. Sampling is performed at the trajectory level rather than at the individual time-step level, so that all samples from the same trajectory remain in the same subset.

#### 2.3.2 Data splitting.

The dataset is split into meta-training, meta-validation, and meta-testing subsets at the vehicle trajectory level. Trajectories from the same vehicle are not allowed to appear in more than one subset. Where recording location information is available, the split is further performed at the recording location level, so that meta-testing tasks come from recording locations that are entirely unseen during meta-training and meta-validation. This ensures that the reported performance reflects adaptation to genuinely new driving conditions rather than memorization of specific vehicles or locations.

The dataset is divided into meta-training, meta-validation, and meta-testing subsets. The meta-validation subset is used for model selection and hyperparameter tuning, while the meta-testing subset is used only for final evaluation.

## 3 Experiments

### 3.1 Data source

The data employed in this study are provided by the highD dataset [29]. It is an open-source naturalistic vehicle trajectory dataset on German highways recorded by drones. As vehicles drive on the right-hand side in Germany, this study mainly focuses on right-hand traffic, in which the driver usually sits on the left side of the car. The dataset includes post-processed trajectories of 110,000 vehicles, including cars and trucks, extracted from drone video recordings at German highways during 2017 and 2018. The recording time of these videos is approximately 16.5 h. The recording frequency of the highD dataset is 25 Hz, and the position error of the recorded tracks is less than 10 cm.

Although the highD dataset contains a large number of vehicle trajectories, not all of them are suitable for lane-changing intention recognition. We therefore apply the following sampling criteria. First, only trajectories with a complete lane-change maneuver or a sufficiently long lane-keeping segment are retained, so that each sample has a well-defined intention label. Second, trajectories with missing or invalid kinematic values over the historical window are excluded, because the Transformer model requires a complete input sequence. Third, only vehicles with complete surrounding vehicle information are considered, so that the selected samples reflect the information available for intention recognition. After applying these criteria, 1426 LK trajectory samples, 325 LCL trajectory samples, and 528 LCR trajectory samples are retained.

To label the retained samples, we follow the lane-changing intention definition in [24]. The lane-crossing event is defined as the moment when the vehicle center crosses the lane marking. For each sample, the intention label is assigned based on the vehicle’s state at the recognition time step, which is set shortly before the lane-crossing event. Specifically, a sample is labeled as LCL or LCR if the vehicle is approaching the lane marking and subsequently completes a left or right lane change, and as LK if the vehicle remains in its current lane. The input to the model is a historical feature sequence of length W = 15 time steps, corresponding to 0.6 s at the 25 Hz sampling rate of the highD dataset. This window ends at the recognition time step and does not include any future information, so the model recognizes the intention based only on past and current motion states. This design is based on the data characteristics of the highD dataset and the experimental design in [30], which provides sufficient motion information for intention recognition.

The dataset exhibits a clear class imbalance, with LK samples substantially outnumbering LCL and LCR samples. Although the dataset itself is imbalanced, balanced episodic sampling is used during meta-training so that each episode contains an equal number of support samples per class. This mitigates the effect of dataset-level imbalance within each episode. The detailed information of the data samples is shown in Table 1.

**Table 1:** The detailed information of data samples in this study.

	Mean Value	Standard Deviation
xVelocity (m/s)	1.21	31.76
yVelocity (m/s)	0.05	0.36
xAcceleration (m/*s*^2^)	0.11	0.23
yAcceleration (m/*s*^2^)	0.01	0.15
Distance Headway (DHW, m)	58.42	63.52
Time Headway (THW, s/veh)	1.88	1.97

The input features are selected based on three considerations. First, they are readily obtainable through V2V communication in connected vehicle environments, which makes the proposed model compatible with real-time applications. Second, they are strongly correlated with lane-changing intention, as lane changes are typically accompanied by changes in velocity, acceleration, and safety margins. Third, they impose low computational cost, which is important for real-time recognition. The selected features include xVelocity, yVelocity, xAcceleration, yAcceleration, Distance Headway (DHW), and Time Headway (THW), as listed in Table 1.

The data collected in this experiment serve as the information of surrounding vehicles that a single vehicle can acquire via V2V communication. Based on this information, the lane-changing intention of surrounding vehicles is recognized using the model proposed in this study. Following the splitting principle described in Section 2.3, the highD dataset is divided into meta-training, meta-validation, and meta-testing subsets at the vehicle trajectory level, with no vehicle appearing in more than one subset. The meta-training subset represents the historical trajectory information that a vehicle can acquire when it initially establishes communication with other connected vehicles via V2V communication. The meta-testing subset represents the trajectories of surrounding connected vehicles that the vehicle acquires during the subsequent driving process.

### 3.2 Experiment design

In this experiment, our proposed algorithm is compared with Transformer as a baseline model, which is not added to meta-learning algorithm to validate the effectiveness of MAML. Both models share the same structure of Transformer, the same learning rate of Optimization, the same loss function and the same training epoch to ensure the fairness of comparison. Adam optimizer [31] is employed to get a result of an accuracy metric in the experiment. The “categorical_crossentropy” loss function [32] is used to measure the error that occurs between the real label value and the result of the recognition. The peoposed algorithm is also compared with LSTM model and Time Conventional Model(TCN) in the experiment, to show the advantage of Meta-Transformer in lane-changing intention recognition.

Specifically, four classification results are defined and statistically calculated for each intention: true positive (TP, the model correctly detects the target intention), true negative (TN, the model correctly detects the other intentions), false positive (FP, the model detects the other intentions as the target one), and false negative (FN, the model detects the target intention as other intentions).

Because the three intention classes are imbalanced, overall accuracy alone may be dominated by the majority class (LK). To provide a more balanced evaluation, we additionally report macro-averaged precision, recall, and F1. Macro-averaged metrics compute the metric for each class independently and then take the unweighted mean, so that LCL and LCR contribute equally to the final score as LK. In the current experiments, balanced episodic sampling is used so that each episode contains an equal number of support samples per class. Nevertheless, the dataset itself remains imbalanced, and the effect of this dataset-level imbalance is further examined through these macro-averaged metrics in Section 3.4.

The accuracy(Acc) of model is calculated as Eq. (9):


$$\begin{array}{c} Acc=\frac{TP+TN}{TP+TN+FP+FN} \end{array}$$ (9)

The precision(Pr) of model is calculated as Eq. (10):


$$\begin{array}{c} Pr=\frac{TP}{TP+FP} \end{array}$$ (10)

The Recall(Re) of model is calculated as Eq. (11):


$$\begin{array}{c} Re=\frac{TP}{TP+FN} \end{array}$$ (11)

The F1-Score of model is calculated as Eq. (12):


$$\begin{array}{c} F1=2\times \frac{Pr\times Re}{Pr+Re} \end{array}$$ (12)

### 3.3 Parameters setting

To initialize the model, some parameters are specially set in the model. The settings of some important parameters in the experiment are shown in Table 2.

**Table 2:** Model parameters settings.

Parameters	Value
Number of Attention Heads	4
Number of Encoder Layers	2
Meta Learning Rate	0.001
Sample Number for Per-round Learning	5
Training Epoch	5000
Train/test ratio	0.7/0.3
Dropout	0.2
Hidden dimension	32
Hidden space	16
Output dimension	3
Optimizer	Adam
Batch/task size	5 samples per class per round
Number of inner-loop steps	1
Random state for data split	34
Random seeds for repeated runs	42, 123, 456, 789, 1010

As shown in Table 2, the model is trained with only 5 samples per class per round, which defines the few-shot scenario evaluated throughout this study. This setting simulates real-world connected vehicle environments where collecting large-scale labeled lane-change trajectory data is extremely challenging. The model described in this paper is built in Python 3.8.4. The model is implemented by using library Pytorch 2.0.0 [33] and learn2learn 0.2.0 [34].

To assess the computational feasibility of the proposed model for practical connected vehicle deployment, we report the number of parameters, model size, inference latency, memory footprint, and MAML adaptation time in Table 3. The Meta-Transformer contains approximately 137,635 parameters, corresponding to a model size of 0.53 MB. The average inference latency per sample is 1.52 ms on an NVIDIA GeForce RTX 2050 GPU with a batch size of 1. The memory footprint of the model parameters is 0.53 MB. The MAML adaptation time per task is 8.68 ms. These results indicate that the proposed Meta-Transformer is computationally lightweight and suitable for real-time lane-changing intention recognition in connected vehicle environments.

**Table 3:** Computational complexity of the proposed model and the baselines.

Model	Params	Model Size (MB)	Latency (ms)	Memory(MB)	Adaptation (ms)
Meta-Transformer	137,635	0.53	1.52	0.53	8.68
Transformer	137,635	0.53	1.56	0.53	N/A
LSTM	13,411	0.05	0.48	0.05	N/A
TCN	22,028	0.08	1.10	0.08	N/A

### 3.4 Results analysis

The overall recognition performance of the Meta-Transformer, along with the benchmark models (Transformer, LSTM, and TCN), is summarized in Table 4. All results are reported as mean ± standard deviation across five independent runs with different random seeds. The proposed Meta-Transformer achieves the highest scores across all four evaluation metrics: Accuracy (93.5 ± 1.3%), Precision (89.7 ± 1.9%), Recall (94.1 ± 1.1%), and F1-Score (91.6 ± 0.6%). Compared to the standard Transformer, which serves as the baseline without meta-learning, the Meta-Transformer improves accuracy and F1-Score by 8.7 and 10.4 percentage points, respectively, demonstrating the significant effectiveness of integrating the Model-Agnostic Meta-Learning (MAML) algorithm. Furthermore, the Meta-Transformer substantially outperforms the LSTM and TCN models, with accuracy improvements of 13.8 and 17.8 percentage points, respectively. It is worth noting that the standard deviation of the Transformer, LSTM, and TCN models is considerably larger than that of the Meta-Transformer, indicating that meta-learning not only improves the average accuracy but also stabilizes the recognition performance across different random initializations and episodic sampling configurations. This stability is particularly important for practical deployment, where consistent performance is required under varying driving conditions.

**Table 4:** Lane-changing intention recognition performance comparison.

	Acc (%)	Pr (%)	Re (%)	F1 Score (%)
Meta-Transformer	93.5 ± 1.3	89.7 ± 1.1	94.1 ± 1.4	91.6 ± 0.6
Transformer	84.8 ± 5.9	83.9 ± 4.7	84.1 ± 8.7	81.2 ± 8.6
LSTM	79.7 ± 5.8	73.7 ± 11.4	74.1 ± 12.0	75.7 ± 10.6
TCN	75.7 ± 8.3	65.8 ± 15.2	55.9 ± 22.2	57.0 ± 18.4

To gain deeper insights, we analyzed the recognition accuracy for each specific driving intention (LK, LCL, and LCR), as illustrated in Fig 3. The Meta-Transformer consistently outperforms the other models across all three intention categories. Notably, all models achieve their highest accuracy on the LK (lane-keeping) intention, which is expected given that LK samples constitute the majority of the driving maneuvers and have more distinct motion patterns. The most challenging intention to recognize is LCR (lane-changing to the right), for which all models show a slight drop in accuracy compared to LCL. This could be attributed to the asymmetric driving behavior in right-hand traffic or the inherent complexity in distinguishing the initiation of a right lane change from other lateral adjustments. Nevertheless, the Meta-Transformer maintains a significant performance margin over the Transformer, LSTM, and TCN for both LCL and LCR, with improvements of approximately 8–10% over the Transformer. This demonstrates that the meta-learning framework is particularly beneficial for recognizing minority classes, where training data is inherently limited.

**Fig 3:**
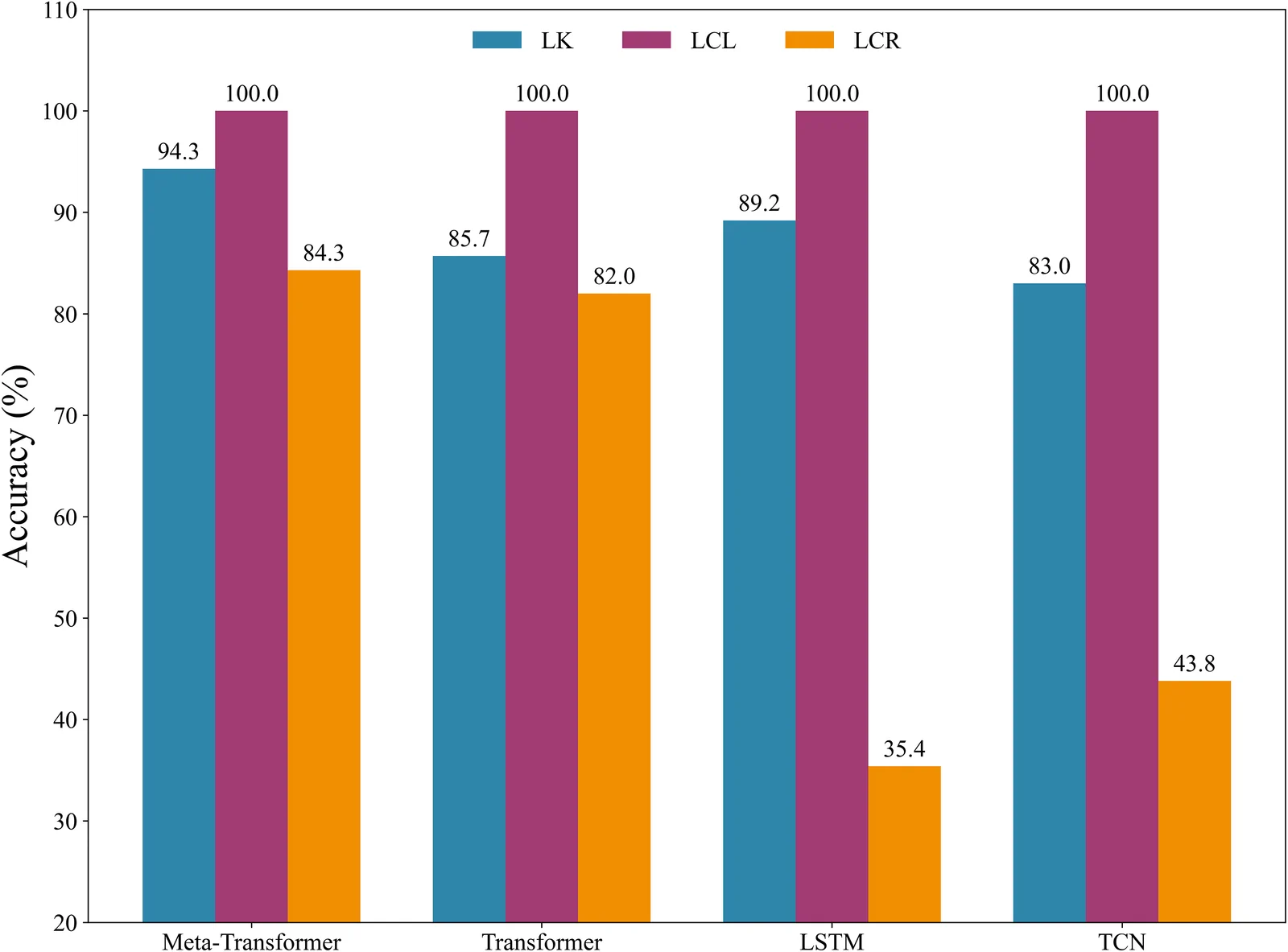
Comparison on LK, LCL and LCR intention recognition accuracy.

The parameter sensitivity of the proposed model is further validated by evaluating its performance under varying time window lengths and training sample sizes, as shown in Figs 4 and 5, respectively.

**Fig 4:**
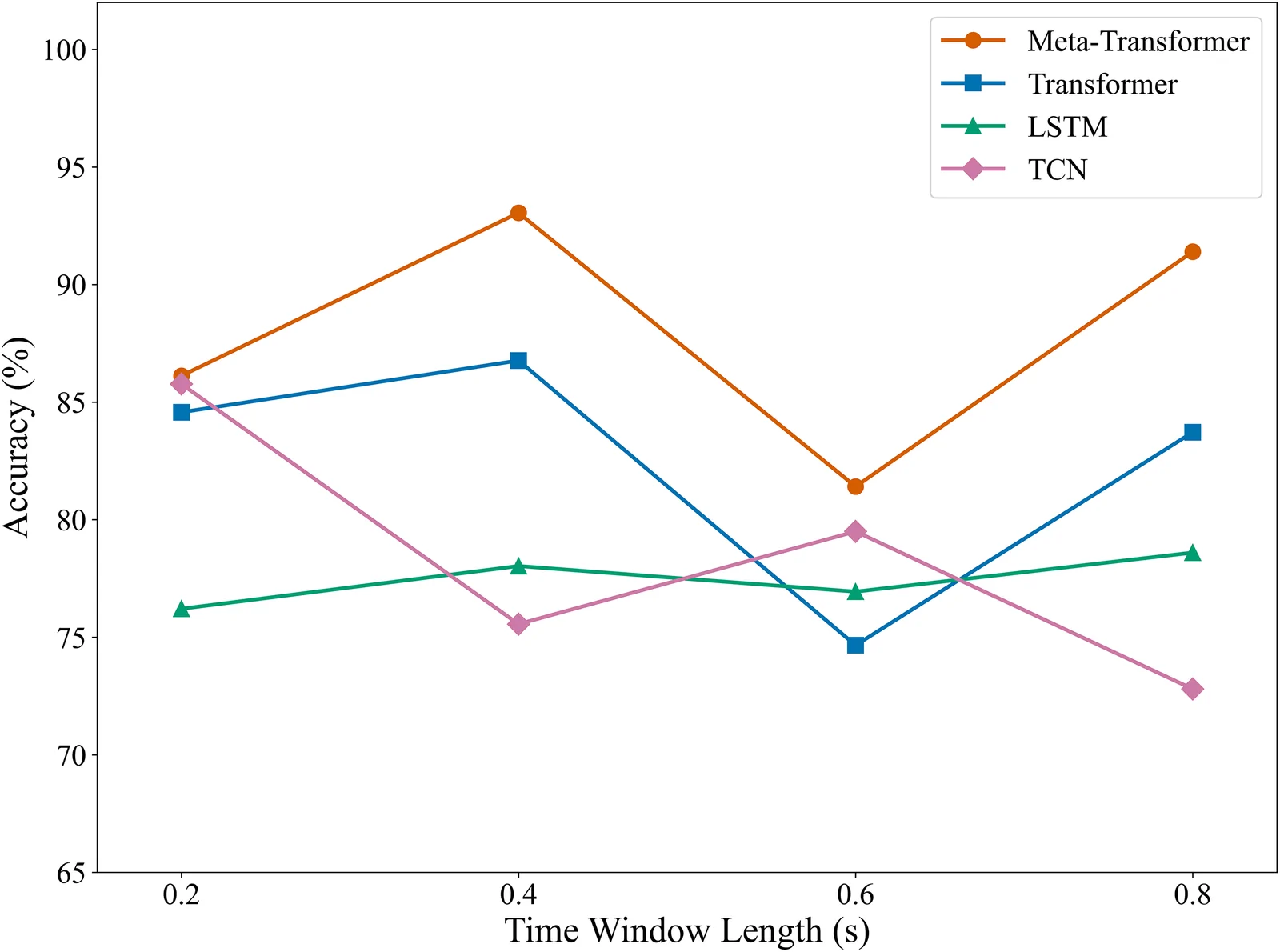
Accuracy comparison with the variation of time window length.

**Fig 5:**
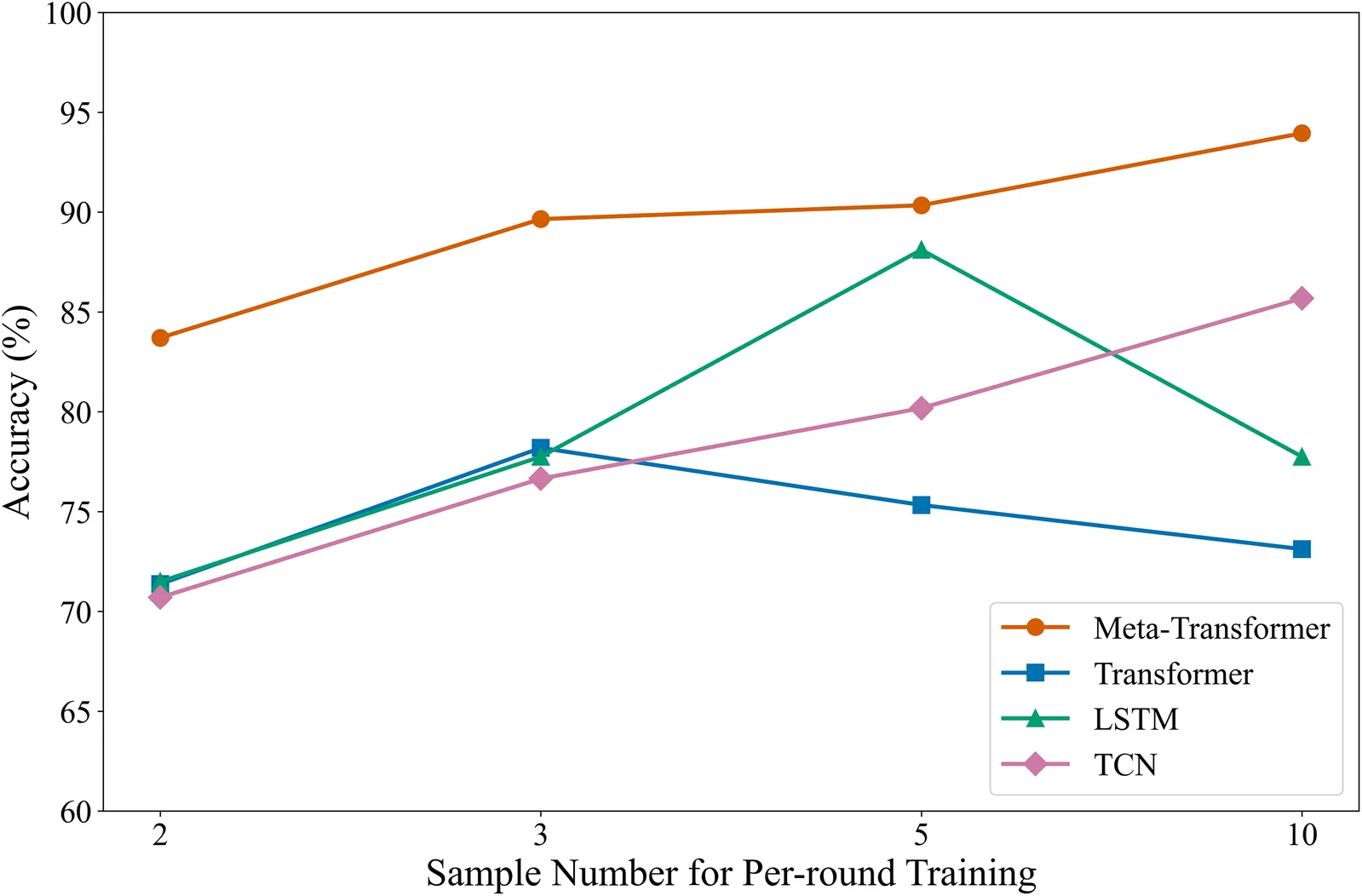
Accuracy comparison with the variation of training sample size.

Fig 4 presents the accuracy comparison as a function of the historical time window length. By comparison on accuracy in Fig 4, it is interesting to find that when time window length is 0.2s, Meta- Transformer achieves close accuracy to Transformer and TCN, while Meta-Transformer still achieves 9.7 percentage points higher accuracy than LSTM. As time window length increases from 0.2s to 0.8s, Meta-Transformer gradually shows significantly higher accuracy than other three algorithms. When the time window length is 0.8 s, the Meta-Transformer achieves an accuracy of 91.4% in this run, which is 8.3 percentage points higher than the Transformer.

Fig 5 examines the model’s performance with respect to the number of training samples per round (shot size). As expected, the accuracy of all models increases with the shot size. However, the Meta-Transformer exhibits a much faster learning curve. With only 5 samples per class, it achieves an accuracy close to 85%, while the Transformer’s accuracy is approximately 75%. When the shot size reaches 30, the Meta-Transformer’s accuracy approaches 91%, whereas the Transformer plateaus at around 83%. This stark contrast highlights the core advantage of the meta-learning approach: it enables rapid adaptation to new tasks with very few labeled examples, a property that is indispensable for practical deployment where collecting extensive labeled data for rare lane-change events is prohibitively expensive. This demonstrates that Meta-Transformer can achieve stably higher accuracy than other three algorithms under few-shot training.

To examine how early the proposed model can recognize lane-changing intention, we fix the historical window length at 0.6 s and evaluate the recognition accuracy for lane-change samples grouped by the time-to-lane-change (TTLC), defined as the interval between the recognition time step and the lane-crossing event. Due to the limited number of lane-change events with precise lane-crossing annotations, the samples are grouped into two TTLC intervals: [0, 1.0) s and [1.0, 2.0) s. As shown in Table 5, the accuracy of all models decreases as TTLC increases, which is consistent with the weaker motion cues available earlier before the lane crossing. Specifically, the Meta-Transformer achieves 89.6% accuracy at TTLC ∈ [0, 1.0) s and 84.2% at TTLC ∈ [1.0, 2.0) s. Although the accuracy drops by 5.4 percentage points when the recognition is required one second earlier, the Meta-Transformer still outperforms the Transformer by 1.9 and 3.0 percentage points in the two intervals, respectively. Notably, the performance gap between the Meta-Transformer and the Transformer widens as TTLC increases, indicating that the meta-learning framework is particularly beneficial for early recognition, where the available motion cues are weaker and the labeled data are more limited.

**Table 5:** Accuracy comparison under different TTLC interval.

Time-to-lane-change (TTLC)	0-1 s	1-2 s
Meta-Transformer	89.6%	84.2%
Transformer	87.7%	81.2%
LSTM	82.2%	79.2%
TCN	77.1%	74.8%

Fig 6 shows the confusion matrices from one example among the five independent runs, providing a detailed visualization of the classification errors for each model under the same test set. In this run, the Meta-Transformer (Fig 6a) exhibits a clean diagonal dominance with minimal misclassifications. Most errors occur between LCL and LCR, which is understandable as both involve lateral movement. Notably, only 28 LCL samples and 31 LCR samples are misclassified as LK, indicating a high discriminative power of the proposed model. In contrast, the standard Transformer (Fig 6b) shows notably more confusion, with 47 LCL samples and 59 LCR samples being incorrectly identified as LK. The LSTM (Fig 6c) and TCN (Fig 6d) models perform substantially worse, particularly the TCN, which misclassifies a large proportion of LCL (116 samples) and LCR (136 samples) as LK. This suggests that these baseline models struggle to distinguish lane-changing events from lane-keeping, especially when the lateral motion is subtle. While Fig 6 shows the results from a single run, the averaged results across five independent runs are reported in Table 4. The averaged results are consistent with the pattern observed in Fig 6, confirming that the Meta-Transformer achieves higher overall accuracy and more balanced recognition across all intention classes.

**Fig 6:**
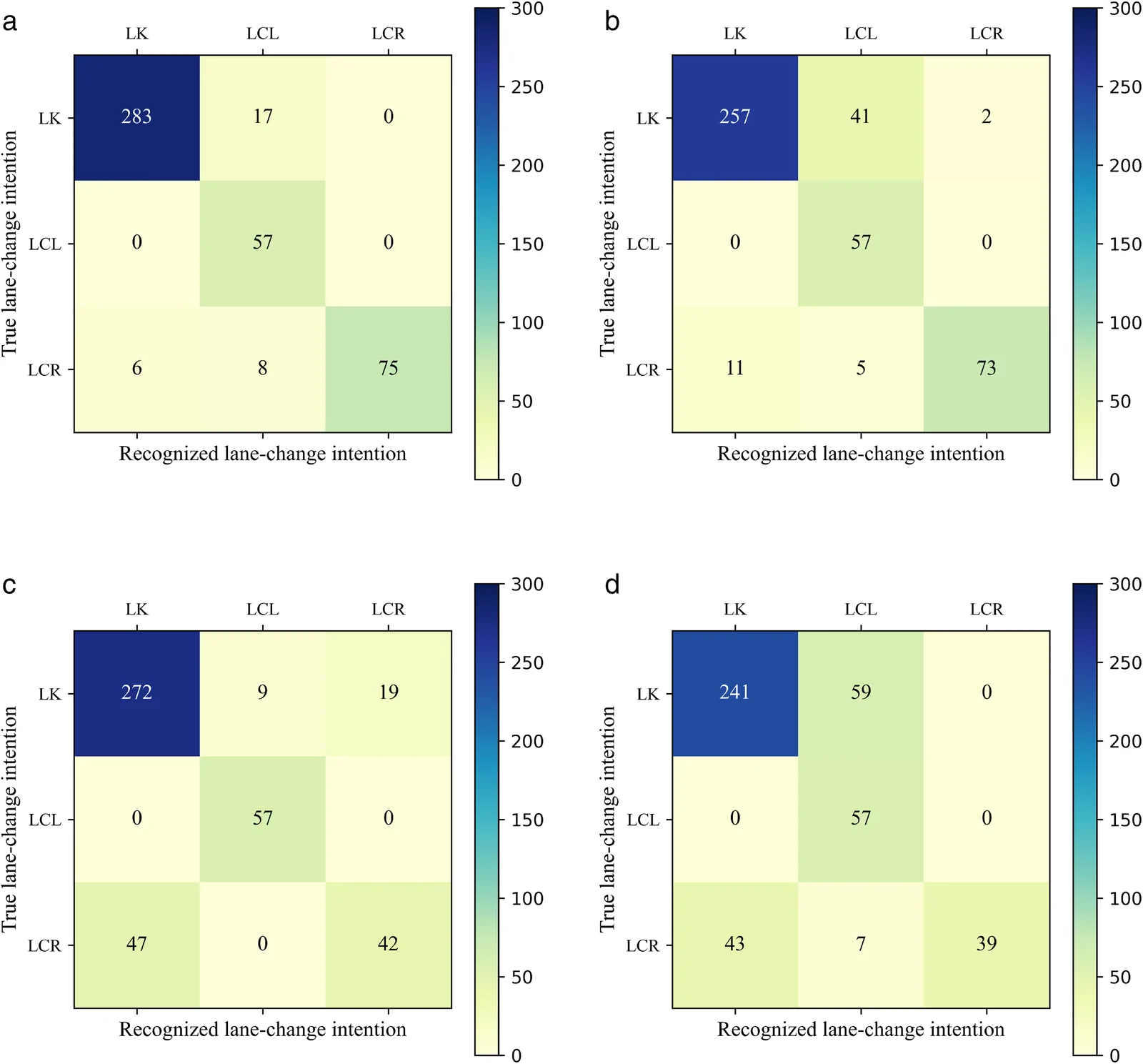
Confusion matrices from one example among the five independent runs for (a) Meta-Transformer, (b) Transformer, (c) LSTM, and (d) TCN.

## 4 Conclusions

This paper focuses on the recognition of lane-changing intention of vehicles and proposes a Meta-Transformer model to solve this problem. The Transformer structure is used to recognize lane-changing intention from sequences composed of specifically selected features. The encoder layer of the Transformer compresses the input feature series into vectors, and the decoder outputs the corresponding recognition results. A MAML algorithm is developed to enable the Transformer model to adapt quickly to limited data and to better solve the recognition problem. During meta-learning, MAML enables the meta-learner to learn the update rules of the Transformer and apply them to update the model parameters. The Adam optimizer is employed to obtain the accuracy metric of recognition, and the categorical cross-entropy loss function is used to measure the error between the real lane-changing intention value and the recognition result.

In the experiment, 2279 vehicle trajectory samples from the highD trajectory dataset are labeled with specific motion labels and used. The Meta-Transformer is compared with the Transformer model, LSTM, and TCN. Experimental results show that the Meta-Transformer achieves 8.7 percentage points higher accuracy than the Transformer model, 13.8 percentage points higher than LSTM, and 17.8 percentage points higher than TCN. These results reveal that the proposed model can achieve high accuracy in lane-changing intention recognition and is better adapted to learning with limited data samples.

Despite the promising results, several limitations should be acknowledged. The current model relies primarily on kinematic features and basic safety indicators, without incorporating driving risk [30], vehicle categories [35], or environmental conditions (e.g., weather and road geometry). Additionally, it assumes constant intention during lane-changing and does not account for intention revisions or aborted maneuvers, which are common in dense traffic. All experiments are conducted on the highD dataset collected on German highways, so cross-dataset generalization remains unknown. The connected vehicle setting in this study is also idealized. The highD dataset is collected by drones and then treated as information available through V2V communication, which does not model realistic communication effects such as latency, packet loss, measurement uncertainty, or incomplete information. In practice, these factors may degrade recognition performance, particularly when the intention must be recognized shortly before the lane crossing. Therefore, the reported performance should be interpreted as an upper bound under ideal communication conditions. Evaluating the proposed model under realistic V2V communication conditions remains an important direction for future work.

Future work will focus on: (1) evaluating the model on additional trajectory datasets and incorporating richer contextual information, including driving risk [30], vehicle types [35], and environmental data, to enhance generalization; (2) extending the model to support continuous intention updating for dynamic traffic scenarios; (3) exploring alternative meta-learning paradigms (e.g., Reptile, prototypical networks) to optimize adaptation efficiency; (4) developing a multi-modal fusion framework that integrates kinematic data with infrastructure-based or visual sensing inputs for more robust recognition; and (5) evaluating the model under realistic V2V communication conditions, including latency, packet loss, and localization errors.
